# RNAslider: a faster engine for consecutive windows folding and its application to the analysis of genomic folding asymmetry

**DOI:** 10.1186/1471-2105-10-76

**Published:** 2009-03-04

**Authors:** Yair Horesh, Ydo Wexler, Ilana Lebenthal, Michal Ziv-Ukelson, Ron Unger

**Affiliations:** 1 Department of Physics of Complex Systems, The Weizmann Institute of Science, Rehovot, Israel; 2 Microsoft Research, Microsoft Corporation, Redmond, USA; 3 The Mina & Everard Goodman Faculty of Life Sciences, Bar-Ilan University, Ramat-Gan, Israel; 4 Computer Science Department, Ben-Gurion University of the Negev, Beer-Sheva, Israel

## Abstract

**Background:**

Scanning large genomes with a sliding window in search of locally stable RNA structures is a well motivated problem in bioinformatics. Given a predefined window size L and an RNA sequence S of size N (L < N), the consecutive windows folding problem is to compute the minimal free energy (MFE) for the folding of each of the L-sized substrings of S. The consecutive windows folding problem can be naively solved in O(NL^3^) by applying any of the classical cubic-time RNA folding algorithms to each of the N-L windows of size L. Recently an O(NL^2^) solution for this problem has been described.

**Results:**

Here, we describe and implement an O(NLψ(L)) engine for the consecutive windows folding problem, where ψ(L) is shown to converge to O(1) under the assumption of a standard probabilistic polymer folding model, yielding an O(L) speedup which is experimentally confirmed. Using this tool, we note an intriguing directionality (5'-3' vs. 3'-5') folding bias, i.e. that the minimal free energy (MFE) of folding is higher in the native direction of the DNA than in the reverse direction of various genomic regions in several organisms including regions of the genomes that do not encode proteins or ncRNA. This bias largely emerges from the genomic dinucleotide bias which affects the MFE, however we see some variations in the folding bias in the different genomic regions when normalized to the dinucleotide bias. We also present results from calculating the MFE landscape of a mouse chromosome 1, characterizing the MFE of the long ncRNA molecules that reside in this chromosome.

**Conclusion:**

The efficient consecutive windows folding engine described in this paper allows for genome wide scans for ncRNA molecules as well as large-scale statistics. This is implemented here as a software tool, called RNAslider, and applied to the scanning of long chromosomes, leading to the observation of features that are visible only on a large scale.

## Background

RNA is typically produced as a single stranded molecule which then folds intra-molecularly to form short base paired stem structures while the unpaired regions form loops. This base paired structure is referred to as the secondary structure of the RNA. Base pairs almost always occur in a non-crossing fashion in RNA secondary structure. Informally, this means that if we draw arcs over an RNA sequence connecting base pairs, none of the arcs cross each other. When crossing base pairs occur, they are called pseudoknots. In this paper, pseudoknots are ignored, and therefore no two arcs are allowed to cross. Under this assumption, a model was proposed in [1] to calculate the stability (in terms of free energy) of a folded RNA molecule by adding independent contributions from base pair stacking and loop-destabilization. This model has been shown to provide a good approximation of the forces governing RNA structure formation, thus enabling fairly accurate predictions of real structures by determining the most stable structure of a given sequence and its corresponding free energy, known as the MFE (Minimal Free Energy). Based on this model, O(N^3^) time algorithms have been proposed and implemented for computing the most stable RNA structure and its MFE, where N denotes the length of the sequences to be folded [2,3]; accordingly, various tools for RNA secondary structure prediction were developed. The tools commonly used today are MFOLD [4], Vienna Package [5] and FOLDRNA [6]. Thus, mapping the entire landscape of a genome of length N looking, for example, at the potential to form ncRNA molecules of size L, can be done by a straight forward application of the classical cubic-time RNA folding algorithm with a sliding window of size L over the genome, resulting in total O(NL^3^) running time. Hofacker et al. [7] suggested an elegant algorithm for this task with run-time of O(NL^2^). Here, we suggest a more efficient consecutive windows folder, whose expected time complexity is O(NL), and give an implementation of this engine, called RNAslider. This faster solution makes consecutive windows folding practical on a genome-wide level and we envision several possible applications for this program, especially as part of a search strategy for long ncRNA molecules. As a demonstration we present an intriguing bias in minimal free energy of genomes depending on their reading direction that we observed by using the program. We also show preliminary results of running RNAslider to characterize long ncRNA molecules on a mouse chromosome and discuss this application further.

## Results

### The fast sliding algorithm

The main recursion used by the current RNA folding algorithms is explicated below:

(1) $$W\left(i,j\right)=\min\left\{V\left(i,j\right),\underset{i\leq k<j}{\min}\left\{W\left(i,k\right)+W\left(k+1,j\right)\right\}\right\}$$

Eq. 1, whose time complexity is classically O(N), where N denotes the length of the RNA sequence S to be folded, computes the optimal folding of substring s_i_...s_j_, which is the value of the entry in row i and column j of the main, upper triangular two dimensional N × N dynamic programming table, W. The application of Eq. 1 involves the computation of the two dimensional matrix, V, whose entries are computed via three additional auxiliary equations. We discuss them briefly below and refer the interested reader to [4] for a thorough discussion and time complexity analysis of these additional auxiliary recursions.

(2) $$V\left(i,j\right)=\min\left\{\begin{array}{l} eh\left(i,j\right),\\ es\left(i,j\right)+V\left(i+1,j-1\right)\\ VBI\left(i,j\right),\\ VM\left(i,j\right)\} \end{array}\right\}$$

Eq. 2 computes the optimal folding energy of a substring s_i_...s_j _in which s_i _base pairs with s_j_, where *eh *denotes the energy term for a hairpin closed by positions i and j and *es *denotes the energy term for the stacking of the base pair (i, j) in a stem that is continued by the base pairing of positions i+1 and j-1. For the sake of simplicity, assume that V(i, j) is set to infinity if the base at position i does not pair with the base at position j of the sequence.

(3) $$VBI\left(i,j\right)=\underset{i\leq i^{\prime }<j^{\prime }<j}{\min}\left\{ebi\left(i,j,i^{\prime }j^{\prime }\right)+V\left(i,j\right)\right\}$$

Eq. 3 computes the score of an optimal folding of substring s_i_...s_j _given the energy of the bulge (ebi) formed at indices (i, i', j', j). (Similarly to the current heavily used folding engines, we assume that the sizes of an internal loop (i' - i) and (j' - j) are bounded by a constant and therefore this term is quadratic.)

(4) $$VM\left(i,j\right)=\underset{i\leq k<j-1}{\min}\left\{W\left(i+1,k\right)+W\left(k+1,j-1\right)\right\}+a$$

where *a *is a constant multi-branch penalty. We note that, for the sake of simplicity of presentation, the current description neglects the contribution for inner pairs of a multiloop and unpaired bases in a multiloop. However, it can easily be extended to apply these contributions without breaking the triangle inequality property on which our algorithm relies.

**DEFINITION 1 **(closed structure).

*A "**closed structure**" over the sequence s_x_...s_*y*_, is a folding in which s_x _pairs with s_y_*.

From Equations 1 and 2 it is easy to see that W(i, j) denotes the optimal (minimal) folding energy that can be obtained by folding the sequence s_i_...s_j _in any possible way. V(i, j), on the other hand, denotes the optimal (minimal) folding energy that can be obtained by a *closed structure *over the sequence s_i_...s_j_. Thus, W(i, j) is computed as the minimum between the lowest folding energy of a "closed structure" V(i, j) and the lowest folding energy of an "unclosed structure", where the energy of each "unclosed structure' can recursively be computed as the sum of two independent parts: W(i, k) + W(k+1, j), where index k is denoted a "partition point". Since the current RNA folding engines bind the size of internal loops (in Eq. 3) to a constant, the main recursion, described in Eq. 1, is the bottleneck recursion. Thus, computing all entries in the upper-triangle of W is classically O(N^3^).

### The consecutive windows folding problem

In this paper, we address a variant of the folding problem, defined below.

**DEFINITION 2 **(Consecutive Windows Folding). *Given a predefined window size L and an RNA sequence S of size N (L < N), the consecutive windows folding problem is to compute the MFE for all L-sized substrings of S*.

The consecutive windows folding problem can be naively solved in O(NL^3^) by applying any of the classical cubic-time folding algorithms to each of the N-L windows of size L. An O(NL^2^) algorithm was described in [7] for the consecutive windows folding problem, which exploits the fact that, when the sliding windows are computed incrementally, in decreasing start-index order, only an L-width diagonal of the original N × N dynamic programming matrix W (for folding the full N-sized sequence), needs to be computed.

An efficient RNA folding algorithm, which computed the optimal folding in expected time complexity of O(N^2^)ψ(N), where ψ(N) was shown to be constant, on average, under standard polymer folding models, was suggested in [8]. This algorithm is briefly reviewed in the next section, and we refer the interested reader to [8] for a more detailed description.

### Review: RNA folding in O(N^2^) expected time

The quadratic-time algorithm for computing an optimal RNA folding utilizes the observation that the main matrix W, which is the final output of the RNA folding recursion, as given in the previous section, obeys the triangle inequality, i.e.

∀*i *≤ *j *<*j*'   *W*(*i*, *j*') ≤ *W*(*i*, *j*)+*W*(*j *+ 1, *j*')

The above claim is used in the next lemma to show that any sum which yields the minimum of Eq. 1 can be reformulated as a corresponding, equal-scoring sum, in which the left term is a closed structure (see Def. 1).

LEMMA 1. *Consider Eq. 1. For every entry W(i, j), if there exists an index k, i ≤ k < j, such that W(i, j) = W(i, k) + W(k+1, j), then W(i, j) = V(i, k') + W(k'+1, j) for some index k' ≤ k*.

The proof of Lemma 1, based on the triangle inequality property of the RNA folding recursion, can be found in [8]. According to Lemma 1, Eq. 1 can be reformulated as follows.

(2a) $$W\left(i,j\right)=\min\left\{V\left(i,j\right),\underset{i\leq k<j}{\min}\left\{V\left(i,k\right)+W\left(k+1,j\right)\right\}\right\}$$

Thus, Eq. 2 could be viewed as a competition between O(N) partition points V(i, k), k = i...j, for the sum: V(i, k) + W(k+1, j) that yields the minimum folding energy. Furthermore, it turns out that some of the competing closed structure partition points dominate others, as becomes clear by the following theorem.

THEOREM 1 [8].

If *V*(*i*, *j*) ≥ *V*(*i*, *k*) + *W*(*k *+ 1, *j*) for some i ≤ k < j then,

∀*j*' > *j V*(*i*, *j*) + *W*(*j *+ 1, *j*') ≥ *V*(*i*, *k*) + *W*(*k *+ 1, *j*').

The redundancies indicated by Theorem 1 can be exploited by maintaining a list of only those partition points V(i, k) that are not dominated by others.

**DEFINITION 3 **(candidate).*A column index j is a candidate in a row i ≤ j iff:*

V(i, j) < V(i, k) +W(k +1, j) ∀ i ≤ k < j.

Note that the above condition is obtained by applying Lemma 1 to the condition

V(i, j) < W(i, k) +W(k +1, j) ∀ i ≤ k < j.

The above definition may be applied to speed up the computation of W(i, j), as follows: Rather than considering all possible O(N) partition point indices for the computation of Eq. 2, only partition points V(i, k) that satisfy the candidacy criterion according to Definition 3 need to be considered. This is formalized in the following equation:

(3a) $$W\left(i,j\right)=\min\left\{V\left(i,j\right),\underset{k\in candidate\_list}{\min}\left\{V\left(i,k\right)+W\left(k+1,j\right)\right\}\right\}$$

Eq. 3 is implemented, in the quadratic-time RNA folding algorithm, via a candidate list that is empty at the start of each row and is extended throughout the left-to-right computation of row i by appending only those partition points V(i, j) which are candidates by Definition 3.

To bind the expected size of the candidate list it is assumed, and later confirmed by benchmark analysis, that the folding predictions generated by the classical RNA folding algorithm follow the standard polymer-folding model. Previous analysis, both theoretical and experimental, has shown that the probability that a closed structure is formed, pairing two positions at distance q monomers apart, is P(q) = $\frac{b}{q^{c}}$ where b = 1 and c > 1. (See [9,10] for self-avoiding random walk (SAW) models for collapsing RNA and other polymers and [11] for a SAW model for DNA denaturation). This fact is explained by modelling the folding of a polymer chain as a self-avoiding random walk in a 2D lattice [12]. In this model, the spatial position of each nucleotide in the original polymer corresponds to a random step within the lattice, where edges of the lattice represent possible transition directions. This walk is called "self avoiding", i.e. it assumes that two bases cannot occupy the same lattice point. The query of interest here is the probability of the q-th step in the walk returning to the origin. The theoretical exponent for the 2D SAW model has been shown to be c = 1.5 [13].

Note that, by Definition 3, each candidate corresponds to a closed structure. Thus, the probability for column j to be a candidate in row i is equivalent to the probability for the optimal folding of s_i_...s_j _to form a closed structure, i.e. *b*·(*j - i*)^-*c*^. Thus, the expected number of candidates in a sequence of length N is ψ(N) = $b\cdot \sum_{i=1}^{N}i^{-c}$. For values c ≥ 1, which is the case in polymer folding, this series is a partial sum of the Riemann Zeta function, defined as $b\cdot \sum_{i=1}^{\infty }i^{-c}$, and is known to converge to a constant. Thus, by applying the candidate list approach described above, the computations performed for each entry in the main dynamic programming matrix W are reduced from O(N) to O(1), yielding the following theorem.

THEOREM 2 [8]. Applying the candidate list algorithm to the folding of an RNA sequence of size N, requires an expected number of O(N^2^) operations.

### An O(NL) algorithm for the consecutive windows folding problem

Hofacker et al. [7] achieved a run-time of O(NL^2^) for the consecutive windows folding problem by observing that, in this application, only an L-width diagonal of the original N × N dynamic programming matrix W (for folding the full N-sized sequence), needs to be computed. In this section we apply this observation to the candidate list algorithm for RNA folding [8] which we briefly discussed above, in order to obtain a faster solution for the consecutive windows folding problem, whose expected time complexity is O(NL). The suggested algorithm also computes an L-width diagonal in the N × N matrix W. The values of this L-width diagonal are computed in decreasing row index order, and then for each row, in increasing column index order. Within this order, the candidate list is emptied out when the computation of each row begins, and then incrementally extended, during the computation of values of this row, by appending only those partition points which are candidates by Definition 3. Each partition point is considered for candidacy once per row, when its column is reached. The algorithm finally returns the vector windowsEnergy, where windowsEnergy[i] denotes the folding energy of S[i ... i+L-1].

The pseudo-code for the algorithm is given below.

Algorithm:

0 windowsEnergy ← NULL

1 for each row i = N-L+1 down to 1 do

2    candidate_list ← NULL

3    for each column j = i up to i + L - 1 do

4       $\text{W}\left(\text{i},\text{j}\right)\leftarrow \underset{\forall k\in candidate\_list}{\min}\left\{\text{V}\left(\text{i},\text{k}\right)+\text{W}\left(\text{k}+1,\text{j}\right)\right\}$

5       if V(i, j) < W(i, j) then

6          W(i, j) ← V(i, j)

7          Append index j to the candidate_list

8       if j = i + L - 1 then

9          windowsEnergy[i] ← W(i, j)

THEOREM 3. The above algorithm computes the optimal folding of N sliding windows, of size L each, in O(NL) expected time and O(N+L^2^) space.

PROOF: The algorithm fills the L-sized diagonal of an N × N matrix, but needs to maintain only an L^2 ^sub-matrix at each step, and thus can be implemented to require O(N+L^2^) space. For each of the N rows, calculating the value for each of the L entries requires the computation of the minimum among ψ(L) candidates, and possibly the addition of a single candidate to the list. Since ψ(L) has been shown to converge to O(1) on average (see Section 1.2), the algorithm spends a constant time on each of the O(NL) entries computed, yielding an O(NL) expected time complexity.

A traceback of an optimal solution is supported at the cost of an additional negligible O(L) term, by maintaining, for each computed entry in W(i, j), a label indicating whether its score was obtained in Eq. 1 from the closed structure term V(i, j) or from one of the partitioning terms V(i, k) + W(k+1, j), and storing the value k in the latter case. Based on this stored information in the computed matrix W, one can recursively recover an optimal traceback for the entire window in O(L) time and in O(L^2^) space, starting from entry W(1, N) [2].

### Testing

**Figure 1:**
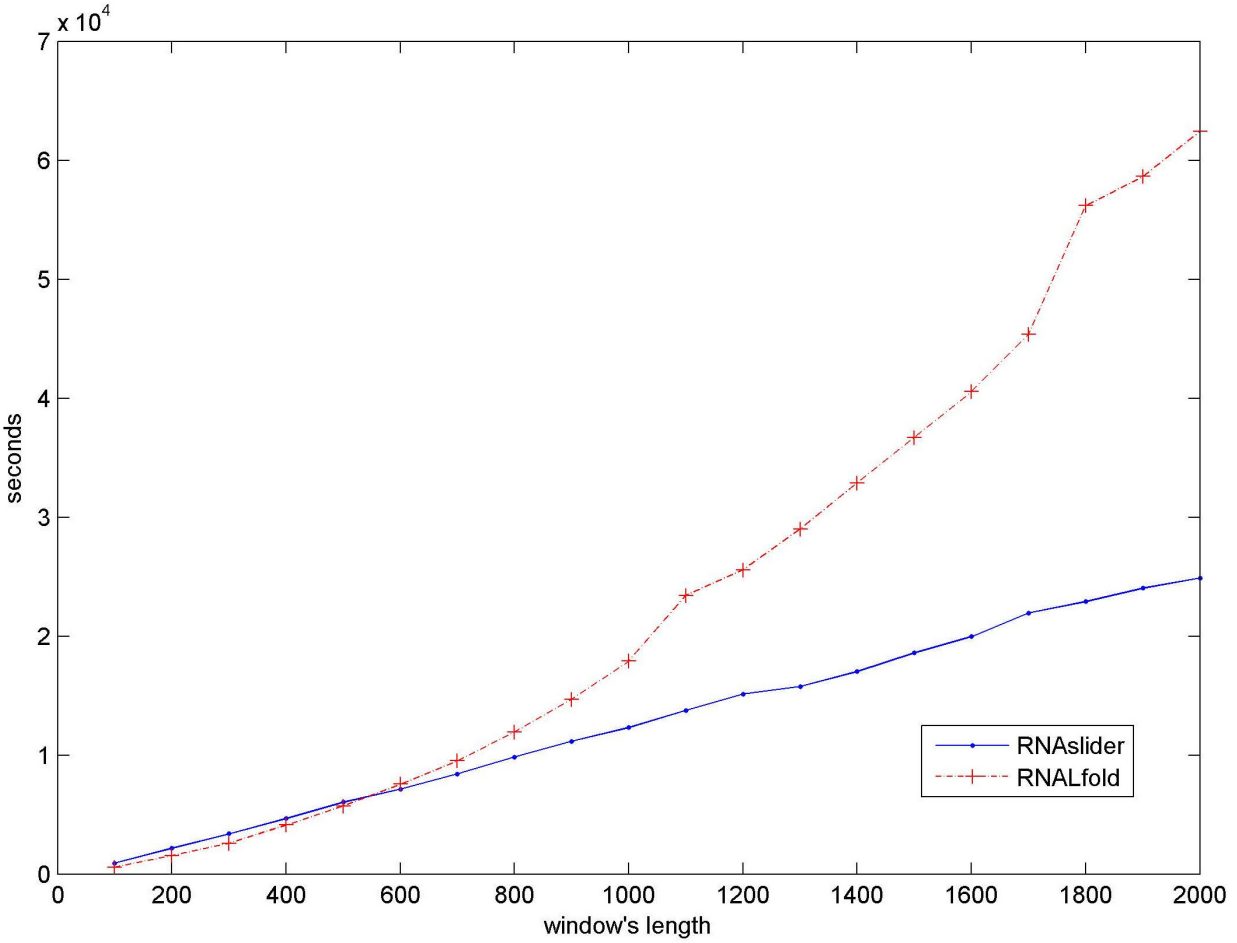
**Measuring the run time of the algorithm**. The first 1,000,000 bps of Oryzias latipes chromosome 9 were scanned with windows of size 100, 200, ..., 2000. As expected, the RNALfold graph fits a quadratic trend line with R = 0.9964, and the RNAslider graph fits a linear trend line with R = 0.999. The runtime measurement was made with an Intel Xeon 3.0 GHz CPU on a Linux machine.

We implemented our Consecutive Windows Folding algorithm in a program called RNAslider. RNAslider is implemented on top of the publicly available code of Michael Zuker and Rune Lyngsψ using its default energy parameters. First, we validated that the running window algorithm gives exactly the same results as running the MFE algorithm on each window separately. Then, we ran it on various sequence lengths and on windows of various sizes. The running time was compared with the running time of RNALfold from the Vienna package, a program that implements the algorithm of Hofacker et al. [7]. Figure 1 demonstrates that the run times of our implementation indeed grow linearly with increasing window sizes, while the RNALfold algorithm has a quadratic running time. We can see that for windows of size of up to 500 bp, the running times are quite similar, with a slight advantage to RNALfold. From size of about 500 bp and up, RNAslider runs increasingly faster than RNALfold. The difference reaches a three fold speed-up for the longer windows of 2,000 bp which are relevant for the analysis of long ncRNA (see Discussion).

## Bioinformatic applications

### Directional Bias – Comparing histograms of MFE distributions

The ability to rapidly calculate the minimal free energy (MFE) values of sliding windows along very large chromosomes enables the probing of MFE landscapes on a genome wide level. First, we explored the MFE landscape of sequences when reading them in the natural 5' to 3' direction, and compared these with the MFEs when reading either in the opposite direction (3' to 5') or on the complementary strand. While the first comparison yielded an intriguing systematic difference between MFEs computed on the "native" sequences versus the "reverse" ones, the comparison of the two opposite strands yielded almost indistinguishable distributions.

**Figure 2:**
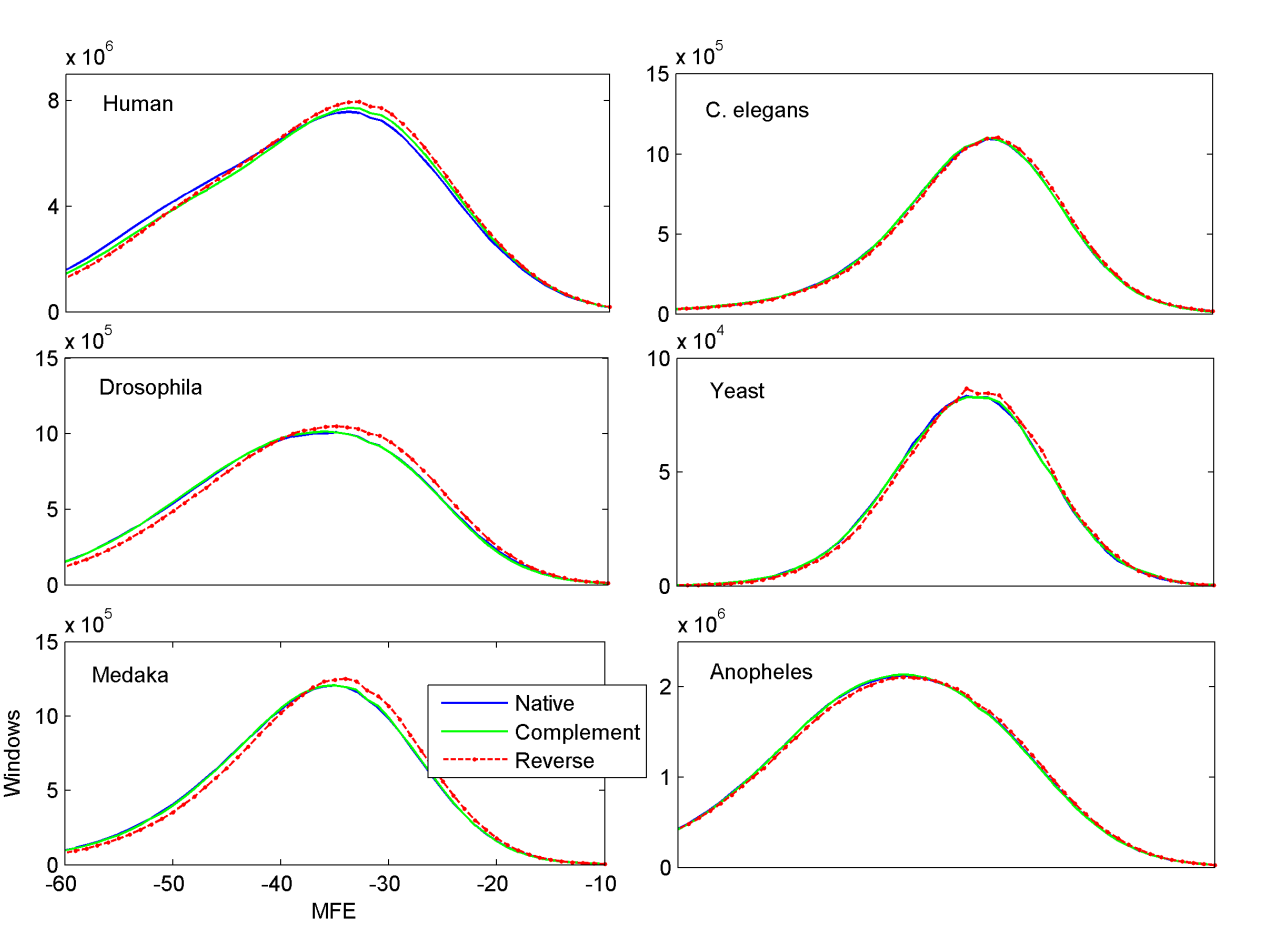
**Genome-wide scans**. Histograms of MFEs for a sliding window of 200 bases calculated by scanning the largest chromosomes of six species. The histograms for the native direction (blue line) and the reverse direction (dashed red line) show a small but clear offset towards lower MFEs (in Kcal/mol units) in the native direction. In contract, the complementary sequence (green line) is almost indistinguishable from the native direction (blue line). The X axis in all six histograms represents the MFE (set to range between -60 to -10), and the Y axis is the number of sliding windows that yielded the respective MFE value.

Figure 2 illustrates histograms based on scanning the largest chromosome from six different species: Human, C. elegans (worm), Drosophila melanogaster (fruit fly), Saccharomyces cerevisiae (yeast), Oryzias latipes (rice fish), and Anopheles gambiae (mosquito). A sliding window of 200 bases was used to scan each chromosome in three directions: native, reverse, and complementary. Visually, the results show that the histograms of the native and reverse complement direction are almost indistinguishable from each other, but the results for folding the reversed sequences show a small offset from the other two histograms, suggesting that sequences that are read from the reverse direction have higher MFEs. This small difference is highly statistically significant. To demonstrate this, and to make sure that the effect is not due to sliding over overlapping and thus dependent windows, we randomly sampled 10,000 non-overlapping sequences of 100 bases taken from human chromosome 1, and found that 6,109 sequences had more stable predicted structures (i.e. got lower MFE) in their native direction, 3,661 sequences preferred the reverse direction, and 230 sequences had the same MFE in both directions. With the null hypothesis of an equal chance to prefer either direction, (counting the 230 indifferent sequences as preferring the reversed direction), a binomial test yielded a p-value < 4.3·10^-110^.

Since each nucleotide monomer of RNA consists of a ribose backbone and phosphate groups attached to the base, it has an inherent asymmetric direction from 5' to 3'. Thus, it is clear for example that the sequence ACGUG is not identical to the reverse sequence GUGCA in its ability to fold into secondary structure. However, it is not clear why, consistently in several genomes, the native coding direction should necessarily have a lower MFE than the reverse direction, especially since only a small portion of genome, especially in higher eukaryotes, is believed to be transcribed and expressed as RNA. In the rest of this section, we further explore this phenomenon.

**DEFINITION 4 **(Directionality Bias): *For a given sequence, S, and a given window-size L, the Directionality Bias (DB) of S by L is the ratio between the number of L-sized sliding windows in S that have a lower MFE when scanned and folded in the native direction, versus the number of L-sized sliding windows in S that have a lower MFE when scanned and folded in the reverse direction*.

For example, in the human chromosome 1, out of 224,998,871 windows of length 200 bases, 148,824,318 (66.14%) yield a lower MFE when folded in the native direction, and only 75,312,478 (33.47%) yield lower MFE when folded in the reverse direction, resulting in a DB of 1.98. We repeated this experiment with the six chromosomes mentioned earlier, and obtained DBs of 1.23, 1.79, 1.32, 1.75, and 1.12 for C. elegans, Drosophila, Yeast, Medaka and Anopheles, respectively.

Note that the DBs reported here were computed in overlapping windows. Similar experiments using different windows sizes (from 50 to 150 nt) yielded similar results.

**Figure 3:**
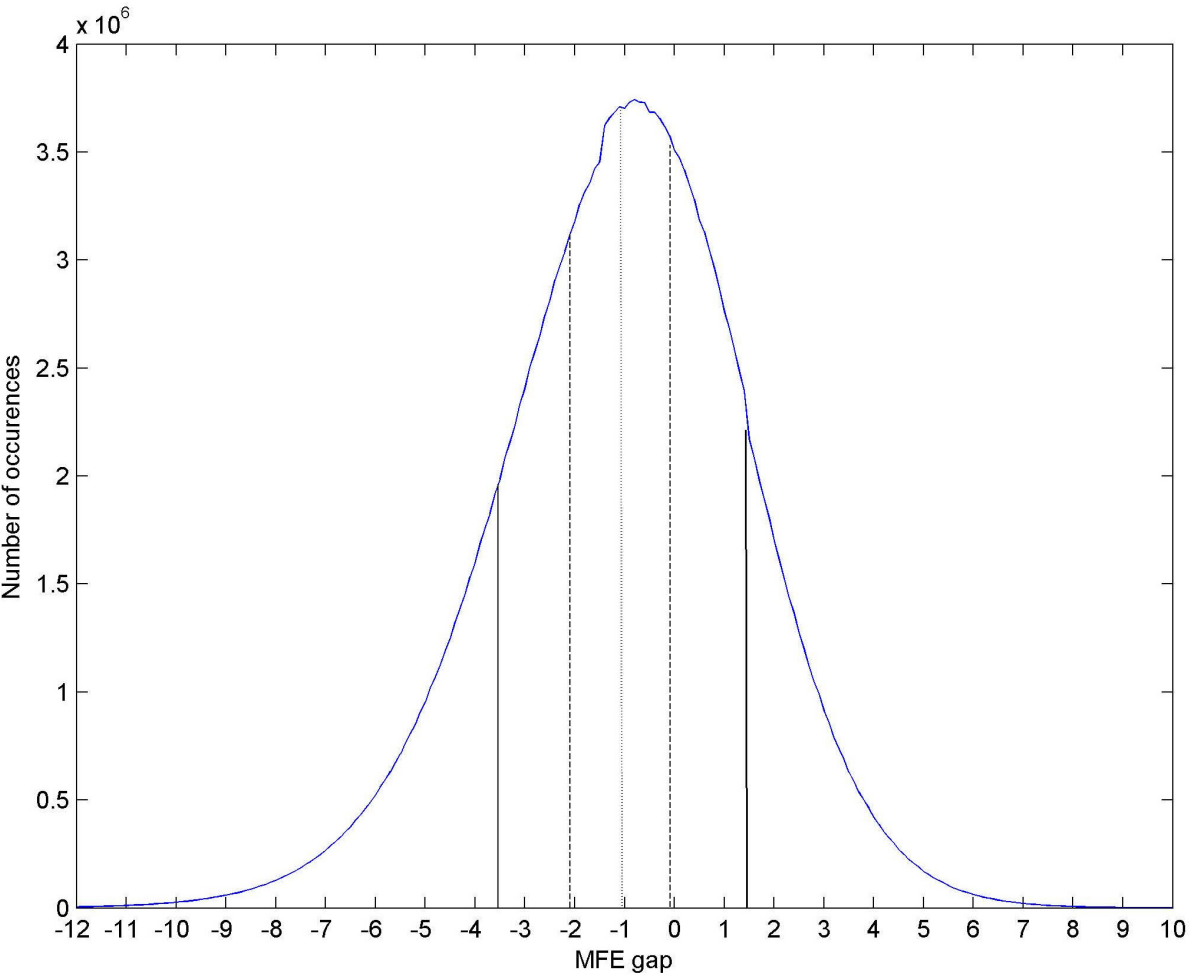
**Difference of MFE histogram**. Human chromosome 1 was scanned with a sliding window of 200 bps twice: first in its native from and then in its reversed form. The MFE gap between each two corresponding windows (i.e. the native window and its reversed) was recorded and is shown with a histogram. The average gap (native MFE – reversed MFE) is -1.06 (dotted line), 34.5% of the volume is confined within 1 Kcal/mol (dashed line) and the standard deviation is 2.45 (solid line). The line is smoothed for visibility.

In addition, we checked the magnitude of this effect. For all windows of length 200 of the human chromosome 1, we made an histogram of the differences in MFE between the native and reverse direction. On average the MFE gap is -1.06 Kcal/mol with standard deviation of 2.45. The histogram, shown in Figure 3, demonstrates that while many of the differences are relatively small (34.5% are within 1 Kcal/mol from the average) there are still a significant number of windows in which the difference is high (e.g. 3.08% of the windows have a difference of 6 Kcal/mol or more).

In order to reveal the origin of this phenomenon we did the following experiment. We created random genomes that maintain the same dinucleotide frequencies of real genomes by using the algorithm described in [14]. Then, we reversed the random genomes and compared the DB of real genomes to that of the random genomes. The comparison shown in Table 1 suggests that most of the effect that we observed comes from the dinucleotide frequencies since the ratio between the DB of real genomes and the DB of shuffled genomes is close to 1. In other words, the DB bias that is attributed only to the dinucleotide frequencies (as is in the shuffled sequences) can account for most of the DB in the real genomes.

**Table 1:** The DB ratios for real and shuffled chromosomes.

**Organism**	**Real DB**	**Shuffled DB**	**DB/DB Ratio**
Homo sapience	1.98	1.66	1.19
C. elegans	1.23	1.27	0.97
Drosophila melanogaste	1.79	1.88	0.95
Saccharomyces cerevisiae	1.32	1.38	0.96
Oryzias latipes	1.75	1.94	0.9
Anopheles gambiae	1.12	1.18	0.95

**Figure 4:**
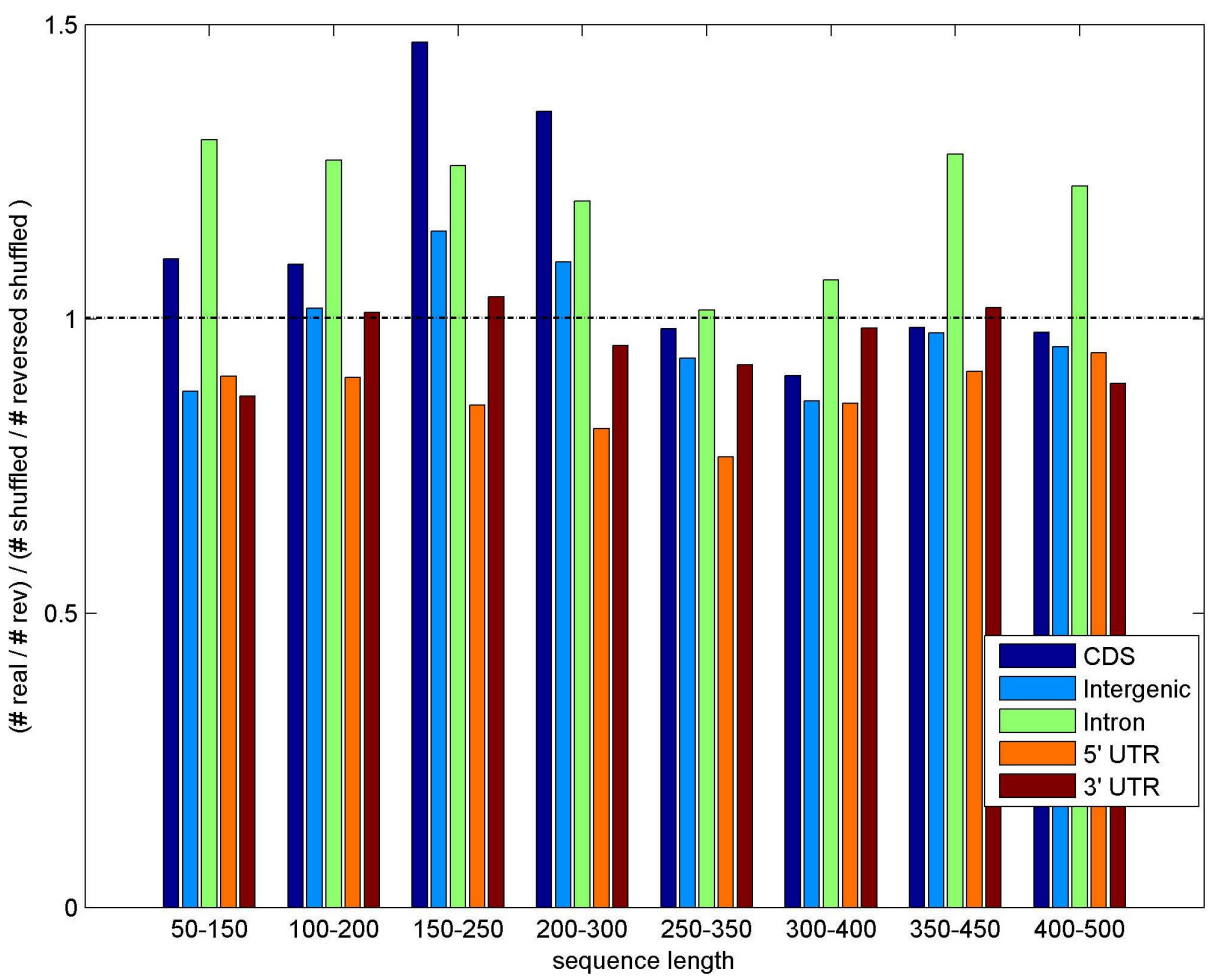
**DB ratio in different genomic regions**. DB ratios (real to shuffled) in different regions of the Drosophila annotated genome, grouped by lengths. We note that in general the coding regions (CDs and introns) have a DB ratio greater than 1 for all windows lengths, while UTR regions (especially the 5' UTRs) have DB ratio less than 1. Intergenic regions seem to have a ratio that is closer to 1.

Despite this fact, we noted that there are some variations in this normalized ratio along different regions in genomes. Figure 4 shows the DB real/DB random ratio over several types of genomic regions of the Drosophila annotated genome (Flybase, version 5.2, Feb 2007). It is interesting to note that in general the coding regions (CDs and introns) have a DB ratio greater than 1 for all windows lengths, while UTR regions (especially the 5' UTRs) have DB ratio less than 1. Intergenic regions seem to have ratio that is closer to 1. These results suggest that for functional regions of the genomes there are additional factors, in addition to the dinucleotide composition, that determine the DB values.

### Analysis of long ncRNA molecules from the mouse genome

We computed the MFE for the entire Chromosome 1 of the mouse of length 197,195,432 bp using sliding windows of 1,000 and 2,000 nt. We then calculated separately the MFE of the windows that contain ncRNA molecules, i.e. we calculated the average MFE of all windows that are contained within each ncRNA molecule. For example, for a molecule of length 2,300, the average is calculated over 301 (2300-2000+1) such windows.

For windows of length 1,000, the genome average MFE is -220.4 Kcal/mol and the average MFE of the 585 ncRNA molecules of size longer than 1,000 (calculated as the average of all windows of length 1,000 that are contained in each molecule) is -231.3 Kcal/mol. For windows of 2,000, the average MFE for the chromosome is -457.8 Kcal/mol and the average over the 339 ncRNA molecules longer than 2,000 nt is -471.6 Kcal/mol. The distributions of the MFE values of the ncRNA molecules and the MFE values of the windows along the entire chromosome are statistically different: Using a two sample t-test, the null hypothesis that the two distributions have the same mean and variance can be rejected with p-values of 5.9·10^-12 ^and 2.5·10^-4 ^for window of 1,000 and 2,000, respectively.

**Figure 5:**
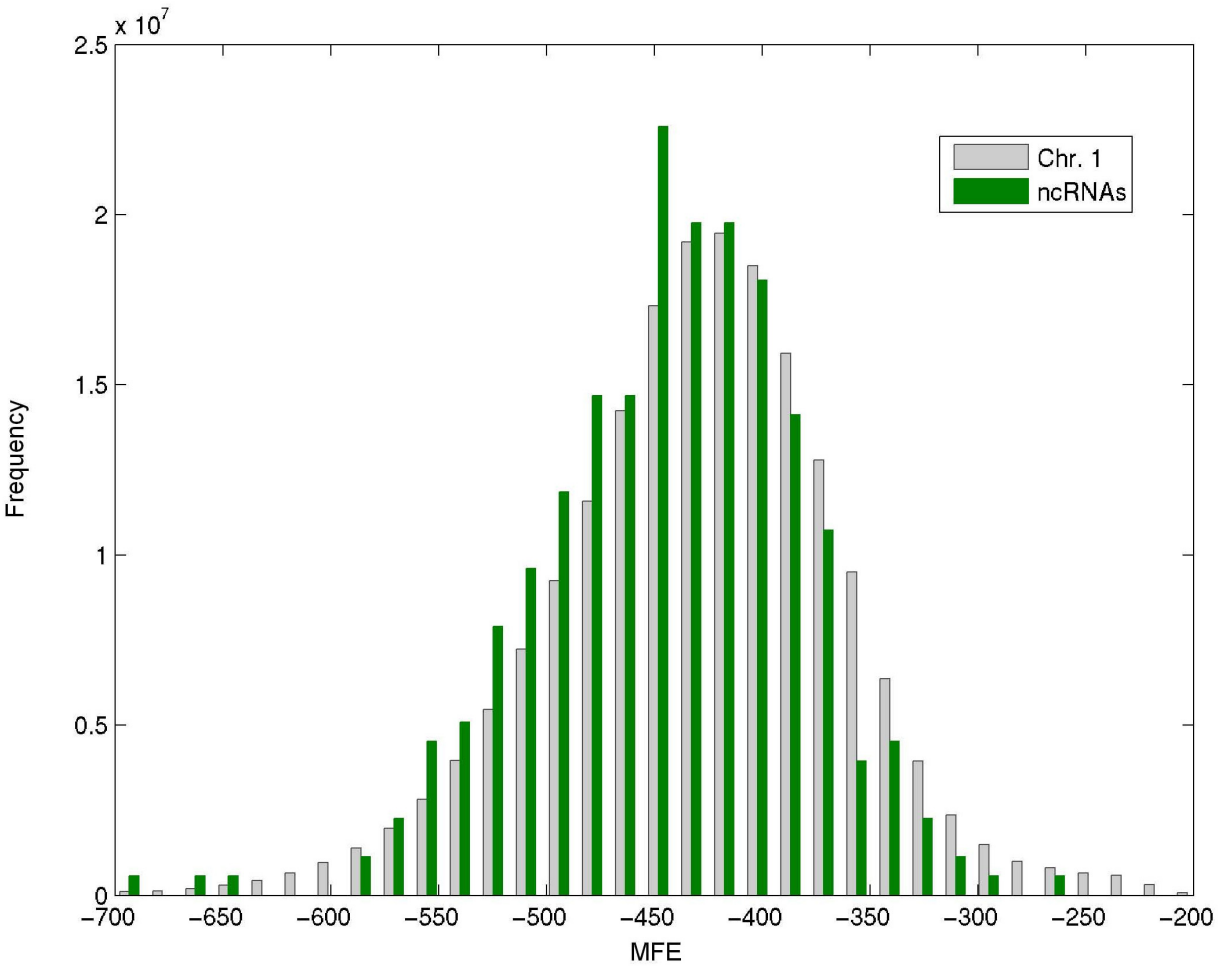
**Distribution of MFE values for long ncRNA molecules in chromosome 1 of the mouse**. The histogram shows the distribution of MFE values for all 197,193,433 windows of size 2,000 nt in chromosome 1 of mouse, compared with the distribution of average MFE values of 339 known ncRNA molecules of length longer than 2,000 nt. For each ncRNA molecule, the average was calculated over all the 2,000 nt windows that are contained within the molecule. The two distributions were normalized to the same size. As can be seen, ncRNA molecules (green) have a tendency to the left; i.e. they have lower MFE values than the rest of the chromosome (gray).

The histograms for the entire chromosome and for the windows that contain the 339 ncRNA molecules with length over 2,000 are shown in Figure 5. It is apparent that the population of the MFE values for the ncRNA molecules is shifted towards the lower MFE values.

We also computed the average MFE of ncRNA molecules of length between 1,000–2,000 nt, using a window of 2,000 nt. In this case we calculated the average MFE over all windows in which the entire molecule is contained. For example, a molecule of length 1,700 nt is fully contained within 301 windows of length 2,000. Although each one of these windows contains regions that are outside the molecule, the average MFE for the 243 ncRNA molecules in this size range was -488 Kcal/mol. We noted that the average MFE calculated for molecules shorter than the 2,000 bp window (243 molecules, average MFE of -488 Kcal/mol) is lower than the average MFE calculated for molecules longer than the same size window (339 molecules, average MFE of -471.6 Kcal/mol). A two sample t-test for the two distributions yields a p-value of 0.0028. This observation suggests that there is an advantage in using windows that are longer than the target molecule size and therefore contain the target molecule in its entirety, over using partial windows that do not contain the entire molecule.

**Figure 6:**
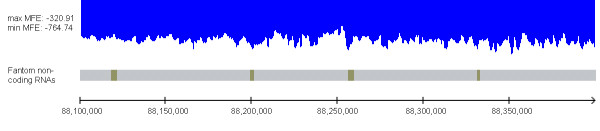
**The MFE landscape and the location of ncRNA molecules**. The MFE landscape (using a sliding window of 2,000 nt) of a region of 300,000 nt from location 88,100,000 to 88,400,000 on mouse chromosome 1. There are four ncRNA molecules of length longer than 1,000 nt in this region. For three out of the four molecules there is a (negative) peak around the genomic location of the ncRNA molecules. Note however that this example is not typical, and there are many peaks in the MFE landscape that do not correspond to ncRNA molecules, and there are also ncRNA molecules that don't reside within or near MFE peaks. The figure was prepared using the Lightweight Genome Viewer available at .

The low MFE for windows associated with ncRNA applies to group averages and thus it is an observation on a statistical level. For some of the ncRNA molecules, there is a correspondence between (negative) peaks of the MFE landscape and the location of individual ncRNA molecules. Figure 6 shows the MFE landscape (using a sliding window of 2,000 nt) of a region of 300,000 nt on mouse chromosome 1, where four ncRNA molecules of length longer than 1,000 nt reside. For three out of the four molecules there is a peak in the genomic location of the ncRNA molecules. We must stress however that this example is not the rule; there are many peaks in the MFE landscape that do not correspond to ncRNA molecules, and there are ncRNA molecules that do not reside within or near MFE peaks.

## Discussion

By combining two algorithmic techniques, the sliding window version of the dynamic programming calculation of MFE (Hofacker et al. [7]) and the utilization of a triangle inequality property of the dynamic programming matrix computed by the RNA folding algorithm (Wexler et al. [8]) we are able to provide a fast algorithm to calculate the MFE of windows along long stretches of DNA or RNA (like chromosomes or genomes) in a time that is linear in the size of the chromosome/genome.

What are the possible applications of such a fast algorithm? As a simple demonstration we first applied our tool to the analysis of the difference in MFE between the native direction of the genome and the reversed direction. As a second application example, we used the tool to compute the MFE for long ncRNA molecules on the background of the MFE landscape of the mouse genome.

### Directional Bias

We have shown that this difference in Directional Bias is largely due to the dinucleotide frequencies, although within a genome there are differences in the DB between different genomic regions. The interplay between the dinucleotide frequency and folding energies is delicate and a source of debate. Seffens and Digby [15] claimed that mRNA sequences tend to have lower free energy than random sequences. Workman and Krogh [16] claim that this tendency originates from the dinucleotide frequency in the genome. A similar position has been taken by Shabalina et al. [17]. It was argued that the periodic pattern of nucleotides, which is created by the structure of the genetic code, influences the dinucleotide frequency which in turn influences folding energies and mRNA secondary structure formation. Our observation that the DB is largely determined by the dinucleotide frequency is consistent with this view. However, there is a kind of a "chicken and an egg" problem here: Does the dinucleotide bias determine folding energy and influence formation of secondary structure, or does the need to form secondary structure influence the dinucleotide composition? We refer the interested reader to a recent paper of Forsdyke [18] that deals directly with this question and highlights the delicate issues in the interplay between dinucleotide composition and folding energies.

### Long ncRNA molecules

Recently, there is an increasing interest in very long non coding RNA sequences. The FANTOM project [19] that is dedicated to the study of the mouse Transciptome has published [20] a list of 34,030 non coding RNA sequences that are transcribed in the mouse. Out of these sequences, 32,364 (95%) are longer than 500 bps, 27,189 (80%) are longer than 1,000 bps, and 15,429 (45%) are longer than 2,000 bps. A very recent study [21] has demonstrated that such long ncRNAs are differentially expressed in the brain and thus are likely to have functional roles, perhaps in memory formation [22]. In [21] it was estimated that about 39% of these transcripts have a conserved secondary structure. It is clear that the field of ncRNA is moving towards studying these long ncRNAs, and for this purpose our RNASlider may turn out to be a helpful component in the analysis toolkit.

We have shown here that the MFE of windows that are contained within long mcRNA molecules is lower, on average, from the MFE of windows that are not associated with ncRNA molecules. Still, as we mentioned above this observation is valid only for large-scale statistics, and it is yet to be determined how it can be exploited, possibly in collaboration with additional RNA analysis tools, to identify long ncRNA molecules. As discussed above, there is evidence to suggest that ncRNA molecules do have lower folding energies than random sequences even when the dinucleotide frequencies are accounted for [23]. Still, since the accuracy of the MFE prediction is known to be limited [24] and a scan of a fixed-length window is bound to add some additional noise, realistically this signal can not be used as a single source for the prediction. An interesting possible direction might be a careful combination of the signal from windows of several lengths. Our observation that MFE values are lower for molecules that are fully contained within a window compared with windows of the same length that cover parts of a longer molecule, suggests that crossing information from different lengths might yield a stronger signal and help to identify approximate borders of suspected molecules. Of course other signals, both experimental (e.g. expression by deep sequencing experiments [25]) and computational (like comparative genomics) must be combined into a comprehensive strategy to identify novel ncRNA molecules.

## Conclusion

We have designed a practical engine that can calculate the RNA minimal free folding energy (MFE) of windows along complete genomes in a reasonable time. For example, calculating all overlapping windows of size 200 of chromosome IV in Saccharomyces cerevisiae, a length of roughly 1.5 million bps, takes about 30 minutes on a single Xeon 3.0 GHz CPU, and the entire human chromosome 1, of roughly 225 million bps, was scanned in about 100 hours. We used this tool to explore an intriguing asymmetry in MFE between the native and the reverse reading frames for both coding and non-coding regions of the genome. We found that the reason for this phenomenon is, largely, the dinucleotide distribution in the genome that apparently prefers, in the native direction, dinucleotides that stabilize folding as reflected in lower MFEs.

This study is only a first demonstration of the kind of large scale questions that could be addressed by our efficient "sliding window folder". As RNA molecules are receiving increased recognition and attention, we believe that a tool that can efficiently scan long sequences and compute their MFEs is likely to be useful for many additional bioinformatic applications, especially when long ncRNAs are analyzed.

## Methods

### Datasets

#### Genomic sequences for the directional bias analysis

The bioinformatic tests were performed on the largest chromosome from six different species: chromosome 1 in Human, chromosome V in C. elegans, chromosome 3R in Drosophila melanogaster, chromosome IV in Saccharomyces cerevisiae (yeast), chromosome 1 in Oryzias latipes (Medaka), and chromosome 2R in Anopheles gambiae. The sequence of the human chromosome was taken from Ensembl version 45 and the sequences of the other organisms were taken from Ensembl version 43.

#### Long ncRNA molecules

Fantom sequences were downloaded from the Fantom3 DB (fantom.gsc.riken.jp). 34,030 sequences were selected as potential non-coding RNAs according to the list taken from . Chromosome 1 of Mus musculus was downloaded from www.ensembl.org (version 47). The locations of the sequences on the mouse genome were determined by MegaBlast. Chromosome 1 of the mouse contains 1,004 Fantom non-coding sequences on the positive strand, however only 686 of them are built from one exon only, and were selected for further analysis.

## Availability and requirements

Project name: RNAslider.

Source code and documentation: 

Operating systems: Unix, Linux, Windows.

Programming language: C.

Restrictions on non-academic use: See license.

## Authors' contributions

YH initiated the study, ran and analyzed the experiments. YW designed the algorithm, and implemented the algorithm in code. IL was responsible on the study of the long ncRNA molecules. MZU designed the algorithm, and took part in the bioinformatic study. RU took part in the bioinformatic study, and coordinated the project. All authors read and approved the final manuscript.
